# Ambulatory blood pressure monitoring before and after resection of catecholamine-secreting pheochromocytoma or paraganglioma

**DOI:** 10.1038/s41371-025-01008-6

**Published:** 2025-03-26

**Authors:** Jordana B. Cohen, Liann Abu Salman, Bonita J. Bennett, Debbie L. Cohen

**Affiliations:** 1 Renal-Electrolyte and Hypertension Division, Department of Medicine, Perelman School of Medicine, University of Pennsylvania, Philadelphia, PA USA; 2 Department of Biostatistics, Epidemiology, and Informatics, Perelman School of Medicine, University of Pennsylvania, Philadelphia, PA USA

**Keywords:** Adrenal gland diseases, Prognosis

## Abstract

Pheochromocytomas/paragangliomas are catecholamine-secreting tumors that are a rare cause of hypertension and associated with high cardiovascular risk. We aimed to evaluate changes in 24-h ambulatory blood pressure (BP) monitoring (ABPM) following tumor resection. Individuals with evidence of pheochromocytoma/paraganglioma prospectively underwent 24-h ABPM and plasma and urine catecholamine measurement 2–3 weeks before and 6–8 weeks after pathologically-confirmed tumor resection. Patients with metastatic disease were excluded. Changes in office, 24-h, daytime, and nighttime mean systolic and diastolic BP and heart rate, 24-h BP and heart rate average real variability, and rates of controlled, sustained, white coat, and masked hypertension were assessed in paired analyses. There were 34 participants who completed 24-h ABPM before and after tumor resection. Mean age was 55 ± 13 years, with 21 (62%) women, median duration of hypertension of 3 years, and 6 (18%) participants with coronary artery disease. Serum and urine catecholamines normalized following tumor resection and participants were prescribed 1 ± 0.2 fewer antihypertensive medications. Office BP declined by a mean 10 ± 16/7 ± 9 mmHg, 24-h BP declined by 8 ± 14/4 ± 10 mmHg, with greater improvement in nighttime (9 ± 18/5 ± 13 mmHg) than daytime (7 ± 14/4 ± 9) ambulatory BP. Systolic BP variability and heart rate variability significantly improved. While the frequency of controlled hypertension increased (4 [12%] to 11 [32%]; *p* = 0.008), there was no significant change in masked hypertension (9 [26%] to 12 [35%]; *p* = 0.366). In conclusion, pheochromocytoma/paraganglioma resection was associated with improvement in 24-h BP and BP variability, particularly nighttime BP. ABPM may be useful following tumor resection to identify patients with unrecognized residual cardiovascular risk.

## Introduction

Catecholamine-secreting tumors are rare tumors arising from chromaffin cells, with an estimated incidence of 2–8 per million and a peak age of occurrence in the third to fifth decade of life [1]. Pheochromocytomas arise from the adrenal medulla and comprise the majority (80–85%) of catecholamine-secreting tumors [2]. Paragangliomas are extra-adrenal and can be divided into sympathetic paragangliomas that arise in the chest, abdomen, or pelvis along the sympathetic ganglion chain, or parasympathetic paragangliomas that arise in the head and neck and are rarely secretory [3]. Classically, patients present with a triad of hypertension, headaches, and diaphoresis; however, the clinical presentation can be quite variable [4]. The clinical manifestations are attributed to catecholamine release, continuously or paroxysmally. Aside from the classical presentation, pheochromocytomas/paragangliomas can also present with chest, abdominal or flank pain, nausea or vomiting, shortness of breath, anxiety, tremors, dizziness, blurry vision, or flushing, and can even be asymptomatic [5–10]. Blood pressure (BP) is classically labile or sustained, but some patients present with normal or low BP. Undiagnosed, these tumors potentially have a high morbidity and can result in hypertensive crises and cerebrovascular or cardiovascular events.

Ambulatory BP monitoring (ABPM) provides a more accurate determination of BP and correlates more closely with cardiovascular risk and mortality than in-office BP measurements [11–16]. The greater prognostic value of ABPM is largely due to its unique ability to identify diurnal patterns of BP control and masked hypertension (i.e., normal BP in the office with elevated BP outside of the office). ABPM is typically performed over a 24-h period with BP readings recorded repeatedly during waking hours and at night while sleeping. Although catecholamine surges are known to contribute to aberrant BP patterns, sparse data exist evaluating ABPM before and after resection of pheochromocytomas and paragangliomas. This study aimed to characterize the circadian patterns of BP on ABPM before and after tumor resection.

## Methods

### Study population

This was a prospective observational study assessing 24-h ABPM before and after complete resection of a pheochromocytoma or paraganglioma. Patients were approached consecutively who had biochemical evidence of pheochromocytoma/paraganglioma based on elevated serum metanephrines at least twice the upper limit of normal of the reporting laboratory and a mass identified on imaging that was suspicious for pheochromocytoma/paraganglioma and amenable to surgical resection. After enrollment and initial pre-operative ABPM, participants were continued in the study for post-operative ABPM if the resected mass was pathologically confirmed as a catecholamine-secreting pheochromocytoma/paraganglioma and successfully fully resected, as determined pathologically. Patients with metastatic disease and incomplete or invalid ABPM were excluded. Participants were enrolled from the University of Pennsylvania Neuroendocrine Hypertension Clinic from January 2014 through December 2019.

### Ethics approval and consent to participate

The study adhered to the principles outlined in the Declaration of Helsinki. The study was approved by the Institutional Review Board at the University of Pennsylvania (Protocol Number 819354). All participants provided written informed consent.

### Measurements

All participants underwent 24-h ABPM 2–3 weeks prior to tumor resection and 6–8 weeks following tumor resection, using the validated Spacelabs 90,217 (Spacelabs Healthcare, Snoqualmie, WA) [17]. For the ABPM protocol, BPs were measured every 20 min during the day and every 30 min at night. ABPM studies were considered valid for analysis if a minimum of 70% of the planned readings were successfully performed [18]. Sleep and wake times were determined by self-report using patient diaries, which were used to determine the time intervals for daytime and nighttime readings.

Hypertension phenotypes accounting for office and ABPM measurements (i.e., controlled hypertension [normal BP in and out of the office], white coat hypertension/effect [elevated BP in the office and normal BP outside of the office], sustained hypertension [elevated BP in and out of the office], and masked hypertension [normal BP in the office and elevated BP outside of the office]) were determined based on the American College of Cardiology/American Heart Association thresholds [19]. Accordingly, normal office BP was defined as ≤ 130/80, normal 24-h ambulatory BP was defined as ≤ 125/75, normal daytime ambulatory BP was defined as ≤ 130/80, and normal nighttime ambulatory BP was defined as ≤ 110/65.

Participants underwent testing of plasma and 24-h urine metanephrines and catecholamines at the visits in which they performed both their pre- and post-operative ABPM. All participants had 24-h urine creatinine levels tested to ensure adherence to the 24-h urine collection.

### Chart review

Demographic information, medical comorbidities, prescribed medications, genetic data, and laboratory values were collected by retrospective chart review. Baseline characteristics were determined at the time of the first ABPM visit that occurred 2–3 weeks prior to tumor resection. Comorbidity-associated variables were not systematically or prospectively collected.

### Statistical analysis

Baseline characteristics were described as mean (standard deviation), median (25th to 75th percentile range), and number (proportion), as appropriate based on data distribution. Unequal variance was assessed and accounted for analytically, as appropriate. Descriptive statistics were performed using the paired t-test, paired Wilcoxon signed-rank test, or paired McNemar’s chi-square test, as appropriate based on data distribution. BP and heart rate variability were determined using the average real variability (ARV), which was calculated as the sum of the absolute differences between consecutive BP readings divided by the total number of BP readings minus one [20]. The average real variability, in contrast to other common metrics of variability such as standard deviation and coefficient of variation, accounts for the sequence of BP measurements and is less sensitive to the magnitude of the BPs and low sampling frequency. Spearman’s correlation was used to estimate nonparametric correlations between catecholamine values and change in 24-h mean SBP after surgery.

Sample size calculations determined that 34 participants were needed to have 80% power to identify a clinically significant, 7 mm Hg decline in mean 24-h SBP at an alpha of 0.05. All analyses were performed using Stata version 18 (Statacorp LP, College Station, TX) with 2‐sided hypothesis testing and an α-level of < 0.05 as the criteria for statistical significance.

## Results

### Cohort characteristics

The study enrolled 39 participants with a pathologically confirmed catecholamine-secreting tumor (pheochromocytoma/paraganglioma); five participants were excluded due to incomplete or invalid ABPM. Baseline demographics of the 34 participants with complete ABPM data are shown in Table 1. Mean age was 55 ± 13 years, 21 (62%) participants were female and 28 (82%) were white. Mean BMI was 26.5 ± 5.3 kg/m^2^ and median duration of hypertension prior to diagnosis was three years. Participants were taking an average of 1.6 ± 0.2 antihypertensive medications at the time of the pre-operative ABPM (prior to many participants having medications adjusted for medically necessary pre-operative alpha blockade). There were 20 (59%) participants who were prescribed an alpha-blocker and 17 (50%) who were prescribed a beta-blocker. Nine (26%) participants had a history of diabetes and six (18%) had a history of coronary artery disease. Germline mutations were present in five (15%) participants.

**Table 1:** Baseline characteristics.

N	34
Age, years	55 (13)
Female sex, n (%)	21 (62%)
Race, n (%)	
White	28 (82%)
Black	5 (15%)
Asian	1 (3%)
BMI, kg/m^2^	26.5 (5.3)
Median (25th percentile, 75th percentile) duration of hypertension, years	3 (1, 10)
Alpha-blocker use, n (%)	20 (59%)
Beta-blocker use, n (%)	17 (50%)
Diabetes mellitus, n (%)	9 (26%)
Coronary artery disease, n (%)	6 (18%)
Germline pathogenic variants, n (%)	
*RET* (p.C634Y, C.1901G > A)	1 (3%)
*NF1* (c.592delG, p.Ala198Hisfs*7)	1 (3%)
*SDHB* (c.137 G > A, p.Arg46Gln)	1 (3%)
*SDHB* (p.R230L)	1 (3%)
*SDHD* (p.W105X)	1 (3%)
Negative	26 (76%)
Missing/not assessed	3 (9%)

Baseline characteristics were collected from the pre-operative clinic visit in which ABPM was placed 2–3 weeks prior to tumor resection. Results are described as mean (standard deviation) or number (proportion) unless otherwise specified.

*BMI* body mass index, *NF1* neurofibromatosis type 1, *RET* rearranged during transfection, *SDHB* succinate dehydrogenase complex iron sulfur subunit B, *SDHD* succinate dehydrogenase complex iron sulfur subunit D.

### Catecholamine measurements before and after tumor resection

Catecholamine measurements pre- and post-operatively are shown in detail in Table 2. Most pheochromocytoma/paraganglioma tumors are predominately norepinephrine- and normetanephrine-secreting, so epinephrine and its metabolite metanephrine are often normal or minimally increased. Pre-operatively, plasma normetanephrine levels were 2–62 (median 5) times higher than the upper limit of normal for the lab (148 pg/mL) and plasma norepinephrine levels were < 1–46 (median 2) times higher than the upper limit of normal for the lab (520 pg/mL). Post-operatively, all catecholamine measurements declined significantly and were within the normal range.

**Table 2:** Serologic and 24-h urine catecholamines before and after tumor resection.

Laboratory Parameter	2–3 weeks before tumor resection	6–8 weeks after tumor resection	Median difference	*P*-value
Plasma metanephrines, pg/mL (normal range ≤ 57)	292 (32, 709)	10 (0, 30)	−282	<0.001
Plasma normetanephrine, pg/mL (normal range ≤ 148)	743 (440, 1179)	86 (64, 146)	−657	<0.001
Plasma epinephrine, pg/mL (normal range 10–200)	114 (26, 294)	19 (6, 30)	−95	<0.001
Plasma norepinephrine, pg/mL (normal range 80–520)	1091 (570, 2029)	390 (331, 610)	−701	<0.001
Urine metanephrine, mcg/24-h (normal range 90–315)	732 (164, 3435)	64 (51, 98)	−668	<0.001
Urine normetanephrine, mcg/24-h (normal range 122–676)	1709 (722, 2838)	314 (225, 460)	−1395	<0.001
Urine epinephrine, mcg/24-h (normal range 2–16)	23 (7, 129)	2 (0, 5)	−21	<0.001
Urine norepinephrine, mcg/24-h (normal range 7–65)	257 (91, 557)	44 (30, 50)	−213	<0.001

All parameters are described as median (25th percentile, 75th percentile).

### BP measurements before and after tumor resection

Office and ABPM measurements obtained 2–3 weeks pre-operatively and 6–8 weeks post-operatively are shown in Table 3. Office systolic BP (SBP) and diastolic BP (DBP) decreased by an average of 10 and 7 mm Hg, respectively (*p* = 0.001; *p* < 0.001). Mean 24-h SBP decreased by an average of 8 mm Hg (*p* = 0.003), with a mean 7 mm Hg decrease in daytime SBP (*p* = 0.005) and 9 mm Hg decrease in nocturnal SBP (*p* = 0.004). Mean 24-h DBP decreased by 4 mm Hg (*p* = 0.022), with a 4 mm Hg decrease in daytime DBP (*p* = 0.026) and a 5 mm Hg decrease in nocturnal DBP (*p* = 0.029). Mean 24-h heart rate decreased by 4 bpm (*p* = 0.017). ARV improved post-operatively with significant decreases in 24–h SBP (−1.4 mm Hg; *p* = 0.007) and heart rate (−1.4 bpm; *p* = 0.036) variability. 24-h DBP ARV decreased but did not reach significance (−0.4 mm Hg; *p* = 0.282).

**Table 3:** Blood pressure and heart rate parameters before and after tumor resection.

	2–3 weeks before tumor resection	6–8 weeks after tumor resection	Mean difference	*P*-value
Antihypertensive medications, N	1.6 (0.2)	0.6 (0.1)	−1.0 (0.2)	<0.001
Office SBP, mmHg	133 (17)	124 (15)	−10 (16)	0.001
Office DBP, mmHg	79 (10)	72 (9)	−6 (9)	<0.001
Office HR, bpm	81 (15)	77 (14)	−4 (15)	0.117
24-h SBP, mmHg	133 (14)	125 (11)	−8 (14)	0.003
24-h DBP, mmHg	78 (11)	74 (10)	−4 (10)	0.022
24-h HR, bpm	78 (11)	74 (9)	−4 (9)	0.017
24-h SBP ARV, mmHg	10.0 (2.7)	8.6 (1.7)	−1.4 (2.7)	0.007
24-h DBP ARV, mmHg	7.6 (1.6)	7.2 (1.9)	−0.4 (2.0)	0.282
24-h HR ARV, bpm	7.3 (3.4)	5.9 (2.1)	−1.4 (3.8)	0.036
Daytime SBP, mmHg	135 (14)	128 (11)	−7 (14)	0.005
Daytime DBP, mmHg	80 (11)	76 (10)	−4 (9)	0.026
Nighttime SBP, mmHg	124 (17)	115 (15)	−9 (18)	0.004
Nighttime DBP, mmHg	70 (13)	65 (11)	−5 (13)	0.029

All values are described as mean (standard deviation) and differences were assessed using the paired t-test.

*ARV* average real variability, *DBP* diastolic blood pressure, *HR* heart rate, *SBP* systolic blood pressure.

**Fig. 1:**
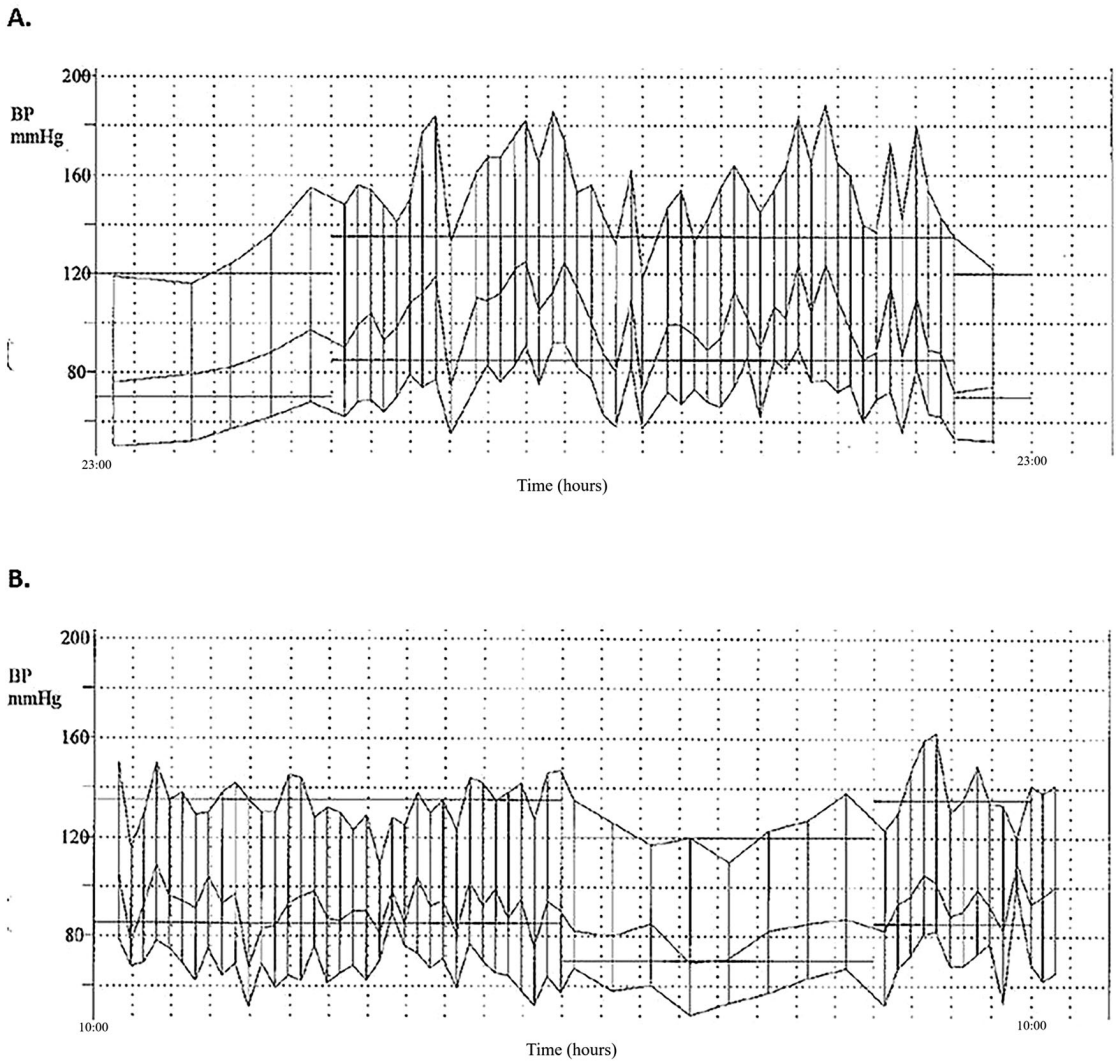
ABPM Before and After Tumor Resection. These are examples of 24-hour ABPMs conducted in the same participant with pathologically confirmed pheochromocytoma/paraganglioma **A** before and **B** after tumor resection.

An example of a typical pre and post-operative ABPM is shown in Fig. 1. This figure demonstrates the pre-operative BP lability during the entire 24-h period, which improved substantially post-operatively.

**Fig. 2:**
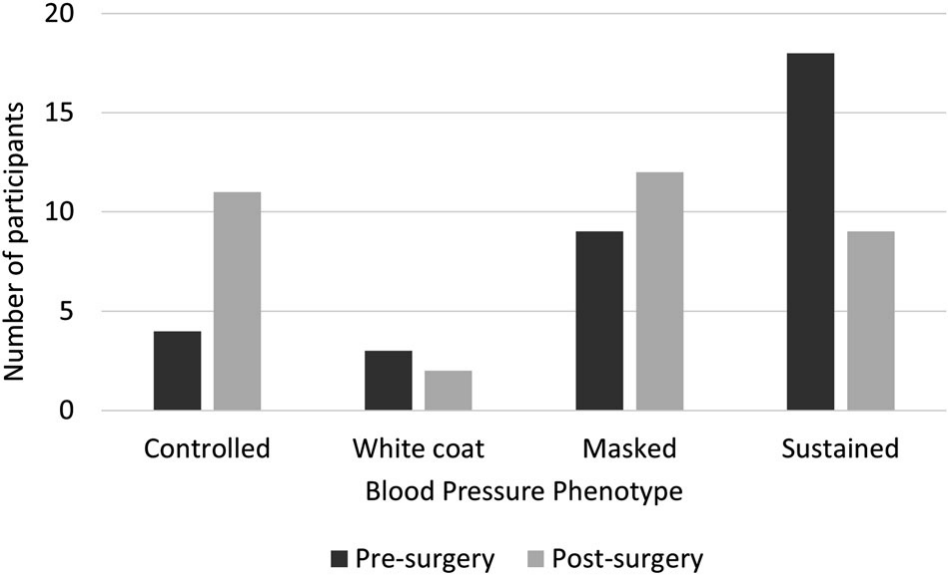
Hypertension Phenotypes Before and After Tumor Resection. Counts of participants with controlled, white coat, masked, and sustained hypertension based on office and ABPM blood pressures 2–3 weeks before and 6–8 weeks after pheochromocytoma/paraganglioma tumor resection.

There were notable changes in hypertension phenotypes following tumor resection (Fig. 2). The number of participants with controlled hypertension increased from four (12%) to 11 (32%; *p* = 0.008) and the number of participants with sustained hypertension decreased from 18 (53%) to nine (26%; *p* = 0.003). Changes in white coat hypertension and masked hypertension were not statistically significant; the number of participants with white coat hypertension decreased from three (9%) to two (6%; *p* = 0.414), and the number of participants with masked hypertension increased from nine (26%) to 12 (35%; *p* = 0.366).

### Correlation of catecholamine measurements with change in BP

At baseline, higher plasma normetanephrine (Spearman’s rho = 0.50, *p* = 0.002; Figure S1) and norepinephrine (Spearman’s rho = 0.60, *p* = 0.001) and urine normetanephrine (Spearman’s rho = 0.60, *p* = 0.001; Figure S2) were significantly correlated with magnitude of decline in mean 24-h SBP. Magnitude of decline in plasma normetanephrine (Spearman’s rho = 0.47, *p* = 0.005; Figure S3) and norepinephrine (Spearman’s rho = 0.56, *p* = 0.003) and urine normetanephrine (Spearman’s rho = 0.77, *p* < 0.001; Figure S4) were significantly correlated with decline in mean 24-h SBP.

### Antihypertensive medication use before and after tumor resection

**Fig. 3:**
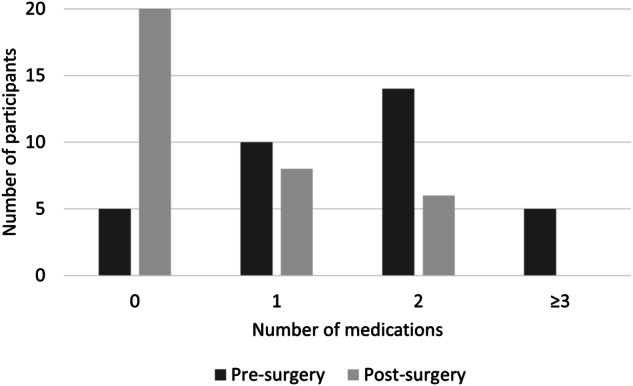
Total Number of Antihypertensive Medications Before and After Tumor Resection. Counts  of distinct antihypertensive medication classes prescribed to participants 2–3 weeks before and 6–8 weeks after pheochromocytoma/paraganglioma tumor resection.

The number of antihypertensive medications pre- and post-operatively are shown in Table 3 and Fig. 3 and specific antihypertensive classes are shown in Table S1. Antihypertensive medication requirement was significantly lower following tumor removal (mean difference in number of antihypertensive medications was −1.0 ± 0.2; *p* < 0.001) with 20 (59%) participants no longer requiring any antihypertensive medications as opposed to five (15%) prior to resection. Of those participants still requiring medication, the number of antihypertensive drugs declined; no participants required three or more drugs and most participants (82%) required either zero or one drug post-operatively.

## Discussion

In this prospective cohort of 34 participants who underwent ABPM before and after resection of pathologically confirmed pheochromocytoma/paraganglioma, we observed that tumor resection was associated with notable declines in office, 24-h ambulatory, daytime ambulatory, and nighttime ambulatory BPs that corresponded with normalization of serum and urine catecholamines and fewer prescribed antihypertensive medications. Furthermore, there was a significant decline in 24-h BP and heart rate variability. Although there was a marked increase in the number of participants with controlled hypertension, there was a non-significant increase in the number of participants with masked hypertension. These findings suggest that some patients may have residual underlying cardiovascular risk following tumor resection that would not be evident using office BP measurements alone.

Our study complements several prior small, cross-sectional, and retrospective studies of ABPM in pheochromocytoma and emphasizes the potential value of greater application of ABPM in pheochromocytoma. ABPM has broadened our understanding of the diurnal and circadian variations in BP and the potential for discordances in BP in the office compared to outside of the medical setting. This has led to many studies demonstrating an association of masked and nocturnal hypertension with an increased risk of adverse cardiovascular events and target organ damage [11–13, 16]. Correspondingly, ABPM has been used in several small studies to evaluate circadian patterns in patients with pheochromocytomas. For example, Dabrowska et al. used 24-h Holter electrocardiography and BP monitoring to evaluate 13 untreated patients with pheochromocytoma matched with 13 untreated patients with primary hypertension. They showed that patients with pheochromocytoma had higher heart rate variability and parasympathetic tone than patients with essential hypertension [21]. To our knowledge, the largest study to date, by Zelinka et al., retrospectively evaluated 54 patients with pheochromocytoma and 108 patients with essential hypertension [22]. Complementary to our observations, the authors found higher 24-h and daytime BP variability, as well as attenuation of circadian BP variability, in patients with pheochromocytoma compared to those with essential hypertension. This carries prognostic value, as BP variability has been established as a risk factor associated with cardiovascular morbidity and mortality [23–25]. Increased BP variability is associated with a higher incidence of hypertensive target organ damage, including left ventricular hypertrophy [26] and early atherosclerosis progression [27]. The mechanism explaining BP variability in pheochromocytomas is likely multifactorial. Catecholamine surges causing paroxysmal hypertension will clearly contribute to increased BP variability. Furthermore, prolonged stimulation of catecholamine receptors leads to desensitization whereby subsequent activation results in receptor downregulation and a diminished response [28]. This downregulation has also been implicated in the mechanism of orthostatic hypotension in patients with pheochromocytoma [29]; another factor leading to increased BP variability.

A few prior, smaller studies have used ABPM to assess physiological changes before and after resection of pheochromocytoma tumors. Minami et al. examined heart rate variability in two patients before and three weeks after tumor resection. Post-surgery, they found a reduction in plasma catecholamine levels and 24-h BP and heart rate [30]. In addition, Bisogni et al. used ABPM to retrospectively evaluate 23 patients with pheochromocytoma/paraganglioma for markers of short-term BP variability and found a significant reduction after tumor resection [31]. Our study is unique in that it includes a larger cohort of patients who all had ABPM performed prior to tumor resection, during a period of high catecholamine secretion, with direct comparison to ABPMs performed 6–8 weeks after resection, when catecholamines returned to normal. It is important to wait for 6 weeks after surgery to assess ABPM, as catecholamines are stored in platelets and can remain high for several weeks after resection [32, 33].

Our study has several limitations. Considering the rare incidence of pheochromocytoma/paragangliomas, the sample size is small, although it is quite larger than that in prior studies evaluating ABPM before and after tumor resection. Regardless, due to the small sample size, we were unable to account for multiplicity across multiple observations. Furthermore, we did not have sufficient statistical power to perform multivariable adjusted analyses to account for differences in baseline characteristics across subjects or to explore potential effect modifiers of BP decline following tumor resection. Nonetheless, the before and after study design was able to limit some of the effects of confounding by using individuals as their own controls. Given the pragmatic nature of the study design, detailed data were not consistently collected to assess target organ damage, such as coronary artery disease.

In conclusion, the long-term cardiovascular effects of catecholamines on BP and subsequent target organ damage in pheochromocytoma/paraganglioma contribute to the high mortality in undiagnosed patients. Our findings reinforce that once a pheochromocytoma/paraganglioma tumor is resected, the effects of high catecholamine levels appear to attenuate. Nonetheless, given the relatively high prevalence of masked hypertension following tumor resection, our findings support the potential utility of out-of-office BP monitoring following tumor resection to identify individuals who may have unrecognized residual cardiovascular risk that would not be captured using office BP measurements alone.

## Summary table

### What is known about the topic

- Pheochromocytomas/paragangliomas are associated with very high cardiovascular risk.
- Ambulatory blood pressure monitoring can provide important insights into cardiovascular beyond office blood pressure measurement.

### What this study adds

- After surgical resection, patients with pheochromocytomas/paragangliomas have high rates of normalization of blood pressure and heart rate and require fewer antihypertensive medications.
- Many patients with pheochromocytomas/paragangliomas have masked hypertension even after surgical resection, suggesting that ABPM may be merited to monitor cardiovascular risk postoperatively in this patient population.

## Supplementary information

Supplemental Material

## Data Availability

Data from this study are not publicly available but will be made available after review and approval of requests by the corresponding author and after the execution of appropriate data sharing agreements.
