# Supplementary material for: Ambulatory blood pressure monitoring before and after resection of catecholamine-secreting pheochromocytoma or paraganglioma
Source: J Hum Hypertens. 2025 Mar 26;39(5):369–75. doi: 10.1038/s41371-025-01008-6 (PMC12069104; doi:10.1038/s41371-025-01008-6)
Supplement: Supplementary file 1 — Supplemental Material [file 41371_2025_1008_MOESM1_ESM.docx]

**Supplement**

**Ambulatory Blood Pressure Monitoring Before and After Resection of Catecholamine-Secreting Pheochromocytoma or Paraganglioma**

Jordana B. Cohen, MD, MSCE; Liann Abu Salman, MD; Bonita J. Bennett, RN; Debbie L. Cohen, MD

**Table of Contents**

[Figure S1. Correlation of Change in 24-hour Mean SBP and Baseline Plasma Metanephrines and Catecholamines. 2](#_Toc190697152)

[Figure S2. Correlation of Change in 24-hour Mean SBP and Baseline Urine Metanephrines and Catecholamines. 3](#_Toc190697153)

[Figure S3. Correlation of Change in 24-hour Mean SBP and Change in Plasma Metanephrines and Catecholamines after Tumor Resection. 4](#_Toc190697154)

[Figure S4. Correlation of Change in 24-hour Mean SBP and Change in Urine Metanephrines and Catecholamines after Tumor Resection. 5](#_Toc190697155)

[Table S1. Antihypertensive Medication Classes Before and After Tumor Resection 6](#_Toc190697156)

# **Figure S1. Correlation of Change in 24-hour Mean SBP and Baseline Plasma Metanephrines and Catecholamines.**

Spearman’s rho p-value was significant for difference in plasma normetanephrine (rho=0.50, p=0.002) and plasma norepinephrine (rho=0.60, p=0.001). All other p-values were >0.05.

Abbreviations: 24h=24-hour; Pl=Plasma; SBP=Systolic blood pressure

# **Figure S2. Correlation of Change in 24-hour Mean SBP and Baseline Urine Metanephrines and Catecholamines.**

Spearman’s rho p-value was significant for difference in urine normetanephrine (rho=0.60, p=0.001). All other p-values were >0.05.

Abbreviations: 24h=24-hour; SBP=Systolic blood pressure

# **Figure S3. Correlation of Change in 24-hour Mean SBP and Change in Plasma Metanephrines and Catecholamines after Tumor Resection.**

Spearman’s rho p-value was significant for difference in plasma normetanephrine (rho=0.47, p=0.005) and plasma norepinephrine (rho=0.56, p=0.003). All other p-values were >0.05.

Abbreviations: 24h=24-hour; Pl=Plasma; SBP=Systolic blood pressure

# **Figure S4. Correlation of Change in 24-hour Mean SBP and Change in Urine Metanephrines and Catecholamines after Tumor Resection.**

Spearman’s rho p-value was significant for difference in urine normetanephrine (rho=0.77, p<0.001). All other p-values were >0.05.

Abbreviations: 24h=24-hour; SBP=Systolic blood pressure

# **Table S1. Antihypertensive Medication Classes Before and After Tumor Resection**

|  | | | |
| --- | --- | --- | --- |
|  | **2-3 weeks before tumor resection** | **6-8 weeks after tumor resection** | **P-value** |
| Antihypertensive medications, N | 1.6 (0.2) | 0.6 (0.1) | <0.001 |
| Alpha-blocker, n (%) | 20 (59%) | 1 (3%) | <0.001 |
| Beta-blocker, n (%) | 17 (50%) | 8 (24%) | 0.013 |
| Calcium channel blocker, n (%) | 7 (21%) | 8 (24%) | 0.655 |
| Angiotensin-converting enzyme inhibitor or angiotensin receptor blocker, n (%) | 4 (12%) | 1 (3%) | 0.083 |
| Diuretic, n (%) | 4 (12%) | 1 (3%) | 0.083 |
| Vasodilator, n (%) | 2 (6%) | 0 (0%) | 0.157 |

Results are described as mean (standard deviation) or number (proportion)
